# Psychological Dimensions of Food Allergy: A Biopsychosocial and Neuropsychological Perspective

**DOI:** 10.3390/nu18101556

**Published:** 2026-05-14

**Authors:** Audrey DunnGalvin

**Affiliations:** Medical Psychology, School of Applied Psychology, University College Cork (UCC), T12 K8AF Cork, Ireland; a.dunngalvin@ucc.ie

**Keywords:** food allergy, quality of life, biopsychosocial model, anxiety, interoception, psychoneuroimmunology, gut–brain axis, family coping

## Abstract

Food allergy is a chronic immune-mediated condition that must be understood not only as a biological disorder but also as a biopsychosocial condition with significant psychological and neurodevelopmental consequences. Beyond the management of acute allergic reactions, individuals living with food allergy experience ongoing threat appraisal, dietary restriction, and social constraints, shaping emotional regulation, cognition, and wellbeing. This review adopts a psychology-led biopsychosocial and neuropsychological framework to examine the mechanisms through which immune activation and food avoidance influence psychological functioning. Drawing on medical psychology, psychoneuroimmunology, and gut–brain research, we explore how threat perception, interoceptive awareness, learning processes, stress physiology, and family context interact to shape emotional and behavioural responses to food allergy. Particular attention is given to the role of risk perception, vigilance, and learned avoidance in driving anxiety and reduced quality of life. By integrating evidence across psychological and biological domains, this review argues for a more comprehensive model of food allergy that recognises the cumulative emotional and neuropsychological burden associated with living with chronic dietary risk. Greater integration of psychological perspectives within allergy care may help support adaptive coping, reduce unnecessary restriction, and improve quality-of-life outcomes for individuals and families affected by food allergy.

## 1. Introduction

Food allergy is a chronic immune-mediated condition that must be understood not only as a biological disorder but also as a biopsychosocial condition with significant psychological and developmental consequences [1]. Prevalence estimates vary depending on diagnostic criteria and population studied, but recent epidemiological studies suggest that food allergy affects approximately 6–8% of children and up to 4% of adults in many high-income countries. Beyond the risk of acute allergic reactions, food allergy imposes ongoing behavioural, emotional, and social demands on individuals and families. Management requires continuous vigilance, strict dietary avoidance, and preparedness to respond to potentially life-threatening reactions [2,3,4].

Historically, food allergy research has been dominated by biomedical perspectives focusing on immune mechanisms, allergen exposure, and clinical management. While this approach has produced critical advances in diagnosis and treatment, it provides only a partial account of the lived experience of food allergy. For patients and families, the condition is not experienced solely through episodic reactions but through the ongoing anticipation of risk and the behavioural adaptations required to manage that risk in everyday life.

A growing body of research demonstrates that the burden of food allergy extends well beyond physical symptoms, affecting emotional wellbeing, quality of life, social participation, and family functioning [3,5,6,7,8]. Quality-of-life impairment is strongly influenced by perceived risk, uncertainty, and the ongoing cognitive–emotional demands of managing safety in everyday contexts, rather than by objective clinical severity alone [3,6,9].

From a medical psychology perspective, food allergy can therefore be conceptualised as a biopsychosocial condition in which immune processes interact with psychological meaning-making, learning mechanisms, and social context. This perspective builds on the biopsychosocial model originally proposed by Engel [1], which emphasises that health and illness emerge from dynamic interactions between biological, psychological, and social systems.

Neuropsychological perspectives further extend this framework by examining how threat detection systems, interoceptive awareness, and learning processes shape emotional responses to food and eating environments. Eating is ordinarily associated with reward, social connection, and emotional regulation; however; in the context of food allergy, food may become associated with danger and uncertainty, with important implications for fear learning, attentional bias, and emotional regulation systems [10,11,12].

At the same time, advances in psychoneuroimmunology and gut–brain research suggest biological pathways through which immune activation, dietary restriction, and stress physiology may interact with mood and cognitive functioning [13,14]. These mechanisms complement, rather than replace, psychological explanations.

The present review extends beyond existing biopsychosocial accounts by adopting a psychology-led integration that emphasises mechanisms of threat appraisal, learning, and interoception as central to understanding the lived experience of food allergy. While prior reviews have acknowledged psychosocial burden, fewer have systematically integrated these processes with neuropsychological and psychoneuroimmunological frameworks.

By explicitly linking cognitive–emotional mechanisms with biological pathways and behavioural adaptation, this review aims to provide a more mechanistically informed account of food allergy as a condition of chronic threat and regulation. In doing so, it offers a framework that generates testable hypotheses regarding the development of anxiety, avoidance, and quality-of-life impairment, and highlights potential targets for psychological and interdisciplinary intervention.

## 2. Methods

This article adopts a structured narrative review approach to synthesise psychological, neuropsychological, and biopsychosocial research relevant to food allergy. While not a systematic review, efforts were made to ensure transparency, breadth, and conceptual coherence in the selection and integration of literature.

Literature searches were conducted across PubMed, Scopus, and PsycINFO for studies published between 2000 and 2025. This timeframe was selected to capture contemporary developments in psychological, neuropsychological, and psychoneuroimmunological research relevant to food allergy. Search terms included combinations of food allergy, quality of life, anxiety, psychological burden, risk perception, interoception, psychoneuroimmunology, gut–brain axis, and family functioning. Searches prioritised peer-reviewed empirical studies, systematic reviews, and key theoretical contributions.

Studies were included if they addressed psychological, behavioural, or neurobiological processes relevant to the lived experience of food allergy. Inclusion criteria comprised peer-reviewed empirical studies, systematic reviews, and key theoretical contributions addressing psychological, behavioural, or neurobiological aspects of food allergy. Research focusing exclusively on immunological mechanisms was included only where it contributed to broader biopsychosocial interpretation.

Exclusion criteria were non-English publications, case reports without broader conceptual relevance, and studies focused solely on acute clinical management without psychological or behavioural components.

Study selection was guided by conceptual relevance to key themes including threat appraisal, learning mechanisms, interoception, stress physiology, and family context. Given the narrative nature of the review, formal quality appraisal was not undertaken.

## 3. Food Allergy as a Biopsychosocial Condition

Food allergy has historically been conceptualised within biomedical frameworks emphasising immune dysregulation, allergen avoidance, and emergency management. While these elements remain essential, such models are insufficient to account for the lived experience of food allergy. From a medical psychology perspective, food allergy can be understood as a biopsychosocial condition in which immune processes, psychological meaning-making, learning, and social context interact dynamically over time [1,3].

The defining feature of food allergy is not only immune reactivity, but the anticipation of harm. Individuals must continuously monitor their environment, interpret bodily sensations, and regulate behaviour in response to perceived threat. These demands recruit cognitive, emotional, and neurobiological systems involved in threat detection, stress regulation, and learning. Psychological outcomes and quality-of-life impairment are often more closely associated with perceived risk, uncertainty, and loss of control than with objective markers of clinical severity [3,6,8].

**Figure 1:**
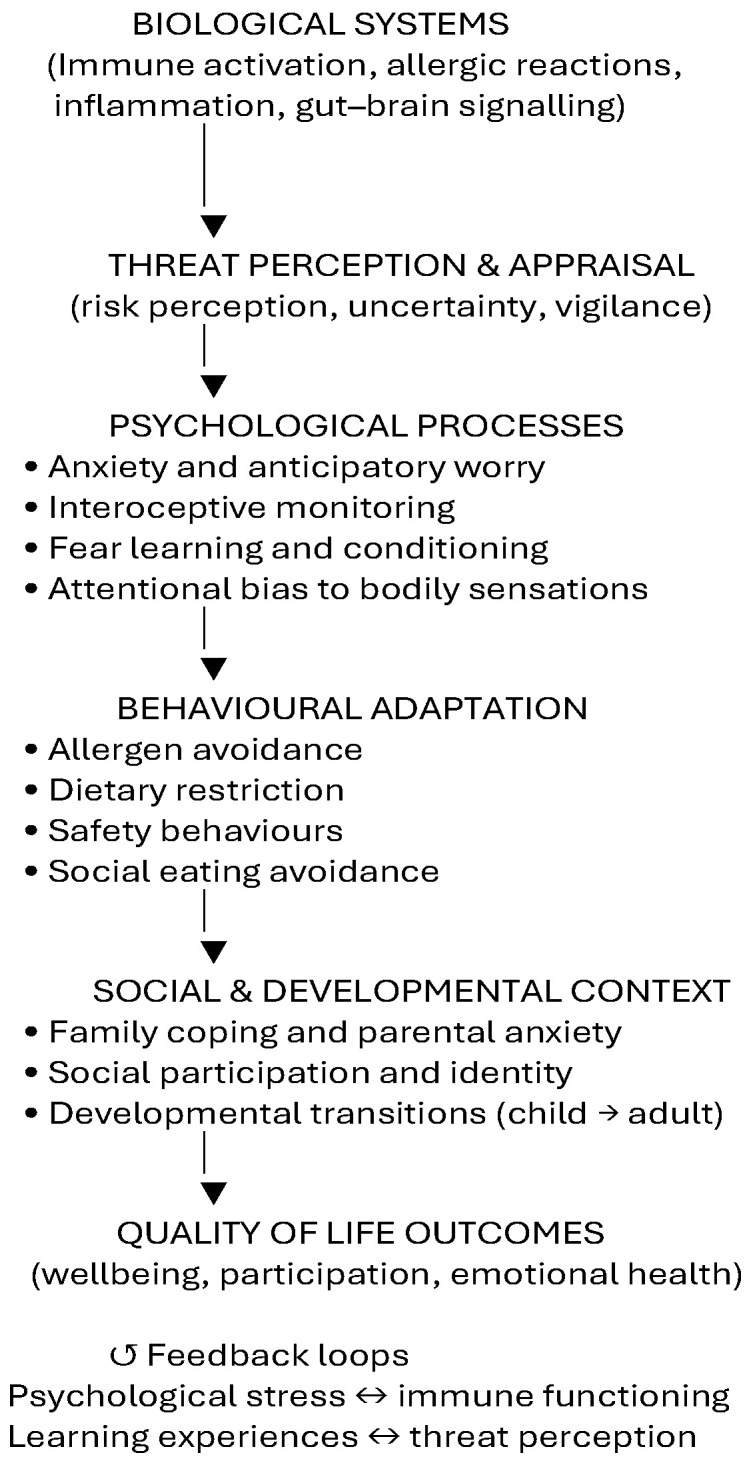
Conceptual biopsychosocial model of psychological burden in food allergy. Food allergy involves dynamic interactions between biological processes (immune activation and gut–brain signalling), psychological mechanisms (threat appraisal, anxiety, and learning processes), behavioural adaptations (avoidance and dietary restriction), and social context (family coping and developmental transitions). These interacting systems shape quality-of-life outcomes and may influence each other through reciprocal feedback loops, including stress–immune interactions and learned threat responses. This model is intended to illustrate hypothesised relationships; it does not imply causal directionality. Arrows represent bidirectional relationships between the biological, psychological, behavioural, and social components.

The relationships between biological, psychological, and social processes in food allergy are illustrated in Figure 1. This model highlights how psychological processes do not merely result from food allergy but actively shape behavioural adaptation, risk perception, and wellbeing over time.

## 4. Threat Appraisal, Vigilance, and Emotional Regulation

### 4.1. Food Allergy as a Condition of Chronic Threat

From early childhood onward, food allergy requires ongoing appraisal of potential danger. Eating, normally a source of pleasure, social connection, and regulation, becomes associated with threat. Neuropsychologically, this places sustained demands on threat detection systems, including the amygdala–insula network involved in fear learning, interoception, and salience detection [10,12].

Unlike time-limited stressors, the risk in food allergy is persistent and unpredictable. Even in well-managed cases, accidental exposure remains possible. This may lead to heightened baseline arousal, particularly in individuals with high trait anxiety or prior adverse reactions [3,6].

### 4.2. Interoception and Misinterpretation of Bodily Sensations

Research on food allergy-related anxiety and quality of life highlights how heightened monitoring of bodily sensations can amplify anticipatory anxiety, even in the absence of allergen exposure, contributing to functional impairment in daily life [3,6,11].

Individuals with food allergy often become highly attuned to internal bodily sensations. While interoceptive awareness is adaptive in detecting early allergic symptoms, heightened interoception may also increase vulnerability to anxiety when ordinary sensations are misinterpreted as signs of an allergic reaction [10,12].

Over time, repeated pairing of bodily sensations with fear may lead to conditioned responses in which anxiety is triggered independently of immune activation. This may help explain why anxiety symptoms in food allergy are not always linked to objective risk.

## 5. Learning, Conditioning, and Fear Generalisation

Food allergy provides a powerful context for associative learning. Allergic reactions are emotionally salient and often frightening, making them potent conditioning events. Classical conditioning processes may lead to fear responses not only to specific allergens, but to broader food categories or eating contexts. While direct evidence in food allergy populations remains limited, findings from fear learning research in related clinical domains suggest that such associative processes may generalise across contexts and contribute to persistent avoidance behaviours.

Operant learning further reinforces avoidance behaviours when anxiety is reduced by restricting food choices or avoiding social eating [15,16]. These learning mechanisms operate within broader social contexts, particularly family systems. Parental modelling of threat appraisal, reassurance-seeking, and behavioural avoidance can shape children’s regulatory strategies and risk perception, especially during early development [17,18,19].

Key psychological and biopsychosocial mechanisms contributing to the burden of food allergy are summarised in Table 1.

Table 1 integrates key psychological mechanisms with their functional role in food allergy, highlighting how these processes interact across biological, psychological, and social domains.

## 6. Dietary Restriction, Reward, and Mood

Research on allergy-specific quality of life has demonstrated that dietary restriction impacts wellbeing not only through nutritional pathways, but through loss of normality, spontaneity, and social participation [3,6,9].

### 6.1. Nutritional Restriction as a Psychological Stressor

Dietary restriction in food allergy is medically necessary but psychologically consequential. Restriction reduces not only nutrient intake but also exposure to food-related reward, pleasure, and social connection. From a neuropsychological perspective, food is a primary reinforcer with strong links to dopaminergic reward pathways implicated in mood regulation.

Evidence from nutritional psychiatry suggests that reduced dietary diversity and micronutrient inadequacy are associated with low mood, irritability, and fatigue [9]. In food allergy, these risks may be compounded by anxiety-driven over-restriction and lack of regular dietetic review.

### 6.2. Food, Mood, and Meaning

Food carries emotional and symbolic meaning beyond its nutritional value. Repeated experiences of exclusion, vigilance, or fear around food may erode positive associations and contribute to anhedonia or reduced quality of life. These processes are best understood psychologically, rather than solely through nutritional metrics.

## 7. Stress Physiology, HPA Axis, and Immune Interaction

Chronic psychological stress activates the hypothalamic–pituitary–adrenal (HPA) axis, with downstream effects on cortisol regulation, immune functioning, and emotional regulation. In food allergy, stress arises not only from reactions themselves but from sustained vigilance and anticipatory anxiety [3,6].

Psychoneuroimmunology research demonstrates bidirectional relationships between stress, immune activation, and mood. Pro-inflammatory cytokines can influence neurotransmitter systems involved in depression and fatigue, while stress-related cortisol dysregulation may exacerbate immune sensitivity [13].

While these mechanisms are supported by broader psychoneuroimmunology research, direct evidence within food allergy populations remains limited.

## 8. The Gut–Brain Axis: Mechanism, Not Master Narrative

The gut–brain axis offers an important mechanistic bridge between diet, immune activity, and emotional regulation. Alterations in gut microbiota composition, gut permeability, and immune signalling may influence stress responsiveness and mood-related pathways via neural, endocrine, and immune routes [14]. While emerging evidence suggests potential links between gut–brain signalling and emotional regulation, direct empirical evidence within food allergy populations remains limited. Accordingly, these pathways should be interpreted as plausible mechanisms rather than established causal processes.

However, within a biopsychosocial framework, gut–brain processes are best conceptualised as one pathway within a broader system, interacting with psychological and behavioural factors. This interpretation is theoretically informed and requires empirical testing within food allergy populations. Dietary restriction, stress, learning history, and family context shape gut–brain functioning, rather than the microbiome acting as a primary causal agent.

Clinically, this supports careful nutritional management and avoidance of unnecessary restriction, while cautioning against reductionist interpretations of gut–brain findings.

## 9. Family Systems and Developmental Pathways

Across multiple studies, the central role of family context has been shown to play a key role in shaping quality-of-life outcomes in paediatric food allergy. Empirical work has shown that parental anxiety and risk perception are positively associated with child anxiety, avoidance behaviours, and reduced participation in everyday activities [18,19]. These findings support family-based conceptualisations of food allergy distress, rather than individual-only models.

Food allergy is embedded within family systems, particularly in childhood. Parental anxiety, coping styles, and beliefs about risk influence children’s emotional regulation, eating behaviour, and autonomy development. Family-level hypervigilance may inadvertently reinforce threat-focused schemas and limit opportunities for adaptive learning.

Across development, psychological challenges evolve. Young children rely on caregivers for co-regulation; adolescents face tensions between safety and independence; and adults may experience cumulative burden related to identity, relationships, and parenting. Longitudinal models are therefore essential.

## 10. Clinical Implications: Towards Integrated Care

A psychology-led biopsychosocial model of food allergy has important implications for clinical practice. Current models of care, which primarily emphasise immunological risk and allergen avoidance, do not fully capture the psychological burden associated with chronic vigilance, uncertainty, and the cognitive–emotional demands of managing safety in everyday life [6,20].

Historically, outcomes in food allergy have been evaluated using biomedical indicators such as reaction severity, immunological markers, and successful allergen avoidance. While these metrics are essential for clinical management, they do not fully reflect the lived experience of the condition. Psychosocial outcomes are often only weakly associated with objective disease severity [3,6,9].

These findings highlight the importance of routinely assessing psychological wellbeing alongside traditional clinical indicators. Screening for anxiety, low mood, food-related fear, and reduced quality of life may help identify individuals and families who would benefit from additional psychological or behavioural support [20]. There is growing evidence that individuals with food allergy frequently report unmet psychological support needs, underscoring the importance of integrating mental health assessment into routine allergy care [20].

Interventions informed by cognitive behavioural principles may be particularly relevant in addressing the mechanisms outlined in this review. In practice, these approaches may include structured work targeting maladaptive threat appraisal, attentional bias to bodily sensations, and safety behaviours. For example, graded exposure to food-related situations, such as eating in social environments, may help reduce avoidance and fear generalisation, while cognitive restructuring can support more adaptive interpretations of perceived risk and bodily sensations. Brief psychoeducational interventions delivered within routine allergy care may also enhance self-management and coping without compromising safety.

Dietetic input also plays a critical role within this framework. Guidance that supports safe dietary expansion, rather than restriction alone, may help reduce unnecessary avoidance and improve both nutritional adequacy and quality of life. This is particularly important given evidence that anxiety-driven over-restriction may contribute to reduced dietary diversity and associated impacts on mood and wellbeing [9,21].

At a systems level, these findings support more integrated models of care in which allergists, dietitians, and psychologists collaborate to address both the medical and psychosocial dimensions of food allergy. Family-based approaches may be especially important in paediatric populations, where parental anxiety, coping style, and risk perception strongly influence children’s emotional adjustment and participation in everyday activities [6,17,18,19].

Such approaches do not undermine safety; rather, they support sustainable self-management and emotional wellbeing. A biopsychosocial approach therefore extends, rather than replaces, biomedical care, providing a more comprehensive framework for understanding and addressing the full burden of food allergy across the lifespan.

## 11. Limitations

Several limitations of this review should be acknowledged. First, as a narrative review, the selection and synthesis of studies are not exhaustive and may be subject to interpretative bias, despite efforts to ensure conceptual breadth and transparency. Second, while the review integrates evidence from multiple disciplines, direct empirical evidence for some proposed mechanisms—particularly neuropsychological and psychoneuroimmunological pathways—remains limited in food allergy populations. As such, some interpretations necessarily draw on findings from adjacent fields.

Third, heterogeneity in measurement approaches, particularly in assessing quality-of-life and psychological outcomes, may limit comparability across studies. Finally, much of the existing literature is cross-sectional, restricting conclusions regarding causal pathways and developmental trajectories. Future research employing longitudinal and experimental designs will be important in testing the mechanisms proposed in this framework.

## 12. Conclusions

Food allergy is best understood as a complex condition in which immune, psychological, and social processes interact over time. By foregrounding psychological and neuropsychological processes, this review highlights how threat appraisal, learning mechanisms, interoceptive awareness, and family dynamics shape the lived experience of food allergy and contribute to its cumulative burden on quality of life. Importantly, psychological responses to food allergy are not merely secondary consequences of the condition but play an active role in shaping behavioural adaptation, coping strategies, and wellbeing across development. Recognising these interactions supports a more integrated model of care in which immunological management is complemented by psychological and behavioural support. Such an approach may help reduce unnecessary restriction, promote adaptive coping, and ultimately improve long-term wellbeing for individuals and families living with food allergy.

## Figures and Tables

**Table 1:** Key psychological and biopsychosocial mechanisms contributing to emotional and behavioural outcomes in food allergy.

Mechanism	Description	Relevance to Food Allergy	Key References
Threat appraisal and risk perception	Cognitive evaluation of potential danger associated with allergen exposure	Persistent perception of risk contributes to anxiety, vigilance, and reduced quality of life	DunnGalvin et al. [2]; Polloni & Muraro [3]
Interoceptive monitoring	Heightened awareness of bodily sensations related to potential allergic reactions	Increased monitoring of bodily sensations may amplify anxiety and lead to misinterpretation of benign sensations	Paulus & Stein [10]; Khalsa et al. [12]
Fear learning and conditioning	Associative learning processes linking foods or eating environments with threat	Allergic reactions may act as conditioning events leading to avoidance of foods or eating contexts	Dunsmoor & Murphy [15]; Olsson et al. [16]
Behavioural avoidance and restriction	Avoidance behaviours aimed at preventing exposure to allergens	While protective, excessive avoidance may lead to reduced dietary diversity and social participation	Herbert et al. [18]; Knibb et al. [20]
Family coping and parental anxiety	Family beliefs, coping strategies, and modelling of risk perception	Parental anxiety and coping behaviours strongly influence child adjustment and food-related anxiety	Knibb et al. [20]; Cushman et al. [6]
Stress physiology and immune interaction	Interaction between psychological stress and immune processes	Chronic stress may influence inflammatory pathways and emotional wellbeing	Dantzer et al. [13]
Gut–brain signalling	Communication between gastrointestinal, immune, and neural systems	Dietary restriction and microbiome alterations may influence mood and stress regulation	Cryan et al. [14]

## Data Availability

No new data were created or analyzed in this study. Data sharing is not applicable to this article.
